# Hyperpolarization Induced by Lipopolysaccharides but Not by Chloroform Is Inhibited by Doxapram, an Inhibitor of Two-P-Domain K^+^ Channel (K2P)

**DOI:** 10.3390/ijms232415787

**Published:** 2022-12-13

**Authors:** Robin L. Cooper, Rebecca M. Krall

**Affiliations:** 1 Department of Biology, University of Kentucky, Lexington, KY 40506-0225, USA; 2 Department of STEM Education, University of Kentucky, Lexington, KY 40506-0001, USA

**Keywords:** lipopolysaccharides, K2P channels, doxapram, *Drosophila*, chloroform, glutamate receptors, membrane potential

## Abstract

Bacterial septicemia is commonly induced by Gram-negative bacteria. The immune response is triggered in part by the secretion of bacterial endotoxin lipopolysaccharide (LPS). LPS induces the subsequent release of inflammatory cytokines which can result in pathological conditions. There is no known blocker to the receptors of LPS. The *Drosophila* larval muscle is an amendable model to rapidly screen various compounds that affect membrane potential and synaptic transmission such as LPS. LPS induces a rapid hyperpolarization in the body wall muscles and depolarization of motor neurons. These actions are blocked by the compound doxapram (10 mM), which is known to inhibit a subtype of the two-P-domain K+ channel (K2P channels). However, the K2P channel blocker PK-THPP had no effect on the *Drosophila* larval muscle at 1 and 10 mM. These channels are activated by chloroform, which also induces a rapid hyperpolarization of these muscles, but the channels are not blocked by doxapram. Likewise, chloroform does not block the depolarization induced by doxapram. LPS blocks the postsynaptic glutamate receptors on *Drosophila* muscle. Pre-exposure to doxapram reduces the LPS block of these ionotropic glutamate receptors. Given that the larval *Drosophila* body wall muscles are depolarized by doxapram and hyperpolarized by chloroform, they offer a model to begin pharmacological profiling of the K2P subtype channels with the potential of identifying blockers for the receptors to mitigate the actions of the Gram-negative endotoxin LPS.

## 1. Introduction

Gram-negative bacterial infections can trigger an immune response in a host from the bacterial secretion of endotoxins (i.e., lipopolysaccharides LPS and repeats-in-toxin RTX) [1,2]. This response is initiated in mammals as the LPS binds to a complex in tissues known as CD14/TLR4/MD2 [3]. The TLR4 receptors are primary receptors which in part are thought to trigger the secondary physiological immune response of cytokine release. Although the inflammatory cytokines enhance the immune response, they also can cause abnormal function in various tissues such as cardiac muscle, skeletal muscle, and neurons [4,5,6,7].

The hunt to find blockers for the receptors to the Gram-negative endotoxin lipopolysaccharide (LPS) has been an area of interest and debate for years. To date there is no known blocker to the LPS receptor complex. The TLR4 protein (i.e., Toll-like receptor 4) is conserved from arthropods to mammals [8] and might offer a potential model for studying possible blockers to the LPS blocker complex.

Even though the Toll receptors were first characterized in *Drosophila melanogaster*, the immune response is not fully mediated by these receptors. The immune deficiency (Imd) signaling pathway appears to be the major receptor-mediated response to LPS exposure [9,10,11]. The peptidoglycan layer and LPS in Gram-negative bacteria then trigger the Imd receptors [12,13,14,15]. However, the distribution on various tissues and regulation in expression levels of the Imd receptors in insects and other arthropods (i.e., crustaceans) have not been identified. Recent research using RNAi approaches [16] in *Drosophila* to block expression to the known Imd receptor PGRC-LC and PGRC-LE, it was demonstrated that LPS responded in the same manner for acute changes as with ones expressing the receptors [17]. Thus, it appears PGRP-LC and PGRP-LE receptors are not responsible for the rapid direct actions of LPS.

As in mammals and larval *D. melanogaster*, the acute and direct action of LPS on tissues has not yet been well studied. The *Drosophila* and crustaceans body wall muscle hyperpolarizes within a second of LPS exposure. Interestingly, it appears that for *Drosophila* muscle, LPS also blocks the glutamate receptors at the neuromuscular junction (NMJ). Recently, it was demonstrated that the channel responsible for hyperpolarizing skeletal muscle, when exposed to the Gram-negative bacterial endotoxin LPS, also appears to be the channel which is blocked by a K2P antagonist doxapram [18].

Likewise, compounds which may interact with K2P channels could potentiate or supplement the action of volatile anesthetics in pathological conditions such in the presence of LPS. The mechanism of action for some anesthetic has been suggested to be due to hyperpolarizing the membrane potential as well as assumptions such as alterations in bilipid membrane fluidity [19] and blocking synaptic transmission [20]. While there are affects in altering ion channel function which decreases electrical conduction, excitability and decrease in presynaptic Ca^2+^ influx, the recent focus with anesthetics is on activation of a particular type of K^+^ channel known as K2P channels. 

The two-P-domain K^+^ channels (i.e., K2P) is one type of channel responsible for the potential action by types of volatile anesthetics such as halothane and chloroform. The family of K2P channels are diverse in their actions and in what stimulates them as well as what blocks them from functioning [21,22]. A K2P channel was first described in yeast and now many types have been identified in other organisms from plants to humans [23,24]. The K2P channels are responsible for maintaining the resting membrane potential of cells. Knowing the gene sequence of the types of K2P channels in various genomes is helpful to narrow an examination to which tissues and cells express the specific subtypes in normal and in pathological conditions. The expression of the K2P channels is altered in cancerous tissues [25] and in other diseases the expression profiles vary in different tissues [25,26]. In healthy tissues, the expression profile of the subtypes and their density also varies among cells within a given tissue.

Modulators of the K2P channels, such as doxapram (trade names-Stimulex or Respiram) which blocks a K2P channel, are used clinically to stimulate respiratory drive when patients are in an induced therapeutic hypothermia after an ischemic stroke or cardiac arrest [27,28,29,30]. Even for infants, doxapram is used to manage apnea and other respiratory disorders [31,32]. This study may have clinical relevance to the general understanding of blocking the acute cellular response to LPS by doxapram and other compounds but at dosages not relevant to mammals and particularly not to humans due to the wide range in responses to LPS and doxapram among animals. It is noted that a lethal dose of LPS in humans is as low as 1 to 2 μg [33] whereas rodents can handle 15 times higher concentrations. Additionally, it is very likely that the varying isoforms of K2P channels, which are somewhat sensitive to doxapram, show differing affinities to doxapram and other compounds. There appear to be 15 known types of K2P channels in humans and 11 known types in *Drosophila* [34,35]. There has been some renaming of the subtypes based on function and pharmacology, but basically there are six subfamilies (TWIK, TREK, TASK, TALK, THIK, and TRESK) [22,36,37,38,39,40,41]. Specifically, TASK-3 misexpression is related with cancer [42] and forms of epilepsy [43]. Interestingly, TASK-1 is activated by halothane and isoflurane [44] and can lead to hyperpolarization of cells. The Tandem-Pore Weak Inward rectifying K^+^ channel (TWIK-1) and the Tandem-pore Acid-Sensing K^+^ channels (TASK-1 and TASK-3) are sensitive to acidic conditions [27,41]. Doxapram inhibits a TASK subtype which is also sensitive to halothane and chloroform [27,45]. More attention is needed to understand the mechanisms of volatile anesthetics and LPS in offsetting the activation potential of K2P channels and blocking of specific K2P channels. Since the skeletal muscle of larval *Drosophila* depolarizes in acidic conditions and to exposure to doxapram [46,47], it is likely that larval muscle expresses a TASK-like K2P channel. There are other specific TASK1/TASK3 inhibitors (i.e., PK-THPP or ML308) which have been identified to have similar effects and at lower concentrations than doxapram [48,49]. Doxapram and PK-THPP were both examined in this study. It is feasible that the larval muscle would also be sensitive to chloroform. Although, chloroform is an unspecific modulator that can impact other classes of ion channels [48,49]. So, effects by chloroform need to be considered with caution.

*Drosophila* may likely serve as a high throughput organism to screen potential actions by various forms of LPS as well as blockers of LPS receptors and to investigate actions of anesthetics on similar targets of LPS such as K2P channels. Further, *Drosophila* are low cost, offer an ease of genetic manipulability, and accessibility to molecular tools. To date, there are no known blockers to the receptors of LPS on cells. If blockers or modulators to LPS responses are discovered, it could save countless lives of people and animals which die every year of bacterial septicemia. Understanding the mechanisms of action of LPS on the function of K2P receptors, some of the same K2P receptors that anesthetics modulate, could lead to clinical treatments of patients in septicemic comas with particular attention to exposure to anesthetics associated with K2P channels. Potentially, doxapram might even serve as a modulator to help alleviate the action of some forms anesthetics if they aid in blocking the same channels. *Drosophila*, as a model, have immensely served in advancing the understanding of biological systems and currently serve a role in clinically relevant research [21,50,51,52,53,54,55].

The purpose of this paper is to present preliminary evidence to support the hypothesis that *Drosophila melanogaster* can potentially be used as a model for testing the effects of LPS on TASK-like K2P channels using the skeletal muscle cell as a model. In addition, the results of preliminary studies on the effects of doxapram as a mediator of LPS on larval *Drosophila* skeletal muscle cells hyperpolarization are presented to support our hypothesis that doxapram is a potential inhibitor of LPS in larval *Drosophila* skeletal muscle cells.

## 2. Results

The results are organized to first present the effects of varying solutions of chloroform and then doxapram on *Drosophila* skeletal muscle cell membrane potential and then to report the findings on varying combinations of compound on membrane potential. The results conclude with findings on the effects of electrical stimulation of the neuromuscular junction of the *Drosophila* skeletal muscle.

### 2.1. Effects of Varying Concentration of Chloroform on Muscle Cell Membrane Potential

**Figure 1:**
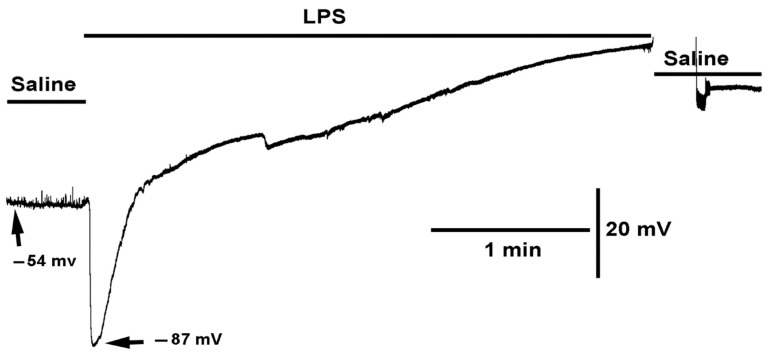
The effect of LPS on membrane potential and synaptic transmission. LPS rapidly hyperpolarized the muscle as well as blocked the receptors to glutamate as the spontaneous quantal events (miniature excitatory junction potentials, mEJPs) were no longer observed even though there was an increased driving force. With prolonged exposure to LPS the membrane potential depolarizes to zero.

Exposure to LPS induced a rapid hyperpolarization of the larval muscle, followed by a gradual repolarization to the original resting membrane potential and then to a depolarized state (Figure 1). Upon removing the LPS containing saline and exchanging with fresh saline, commonly the membrane potential regained some negative potential however the longer the muscle remains exposed to LPS the less likely it will remain healthy even after flushing with fresh saline. As previously demonstrated, LPS blocks the glutamate receptors on larval muscle fibers [56] resulting in the disappearance of the spontaneous quantal postsynaptic events (miniature excitatory junction potentials, mEJPs) as observed during the initial saline exposure. Despite the larger driving gradient for the Na^+^ ions to flow through the inotropic glutamate receptor while the membrane was hyperpolarized by LPS, the mEJPs disappeared. Evoked EJPs were previously shown to be blocked during exposure to LPS [56].

**Figure 2:**
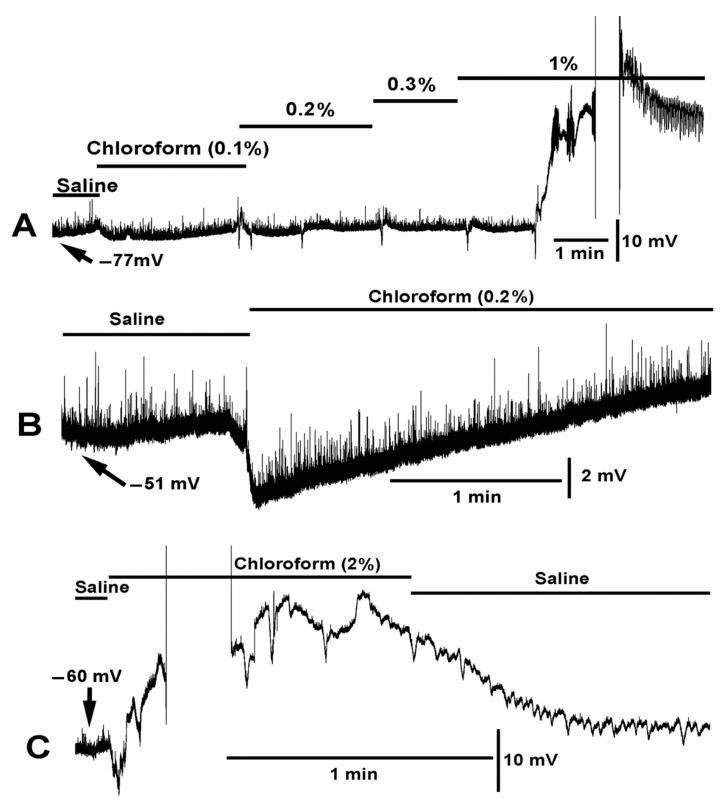
Dose dependent effect of chloroform on membrane potential and muscle contraction. (**A**) Chloroform in saline produced little effect at 0.1% concentration on hyperpolarizing the membrane potential. As the percent of chloroform increased a more pronounced rapid hyperpolarization (<1 s) would occur. Random hyperpolarization occurred during the 0.2% exposure. As the percent of chloroform reached 1% the muscle produced strong contractions, dislodging the intracellular electrode, and would continue to produce waves of contractions as shown at the end of the trace. (**B**) A saline with 0.2% chloroform produced a rapid hyperpolarization followed with a repolarization within 1 min. Note: The miniature excitatory junction potentials (mEJPs) are still present during the hyperpolarization. (**C**) At 2% chloroform, a rapid hyperpolarization occurred followed by strong muscle contraction making it difficult to maintain an intracellular recording. After reestablishing an intracellular recording in the same muscle, the repeated depolarization and hyperpolarization occurred due to muscle producing waves of contractions.

In examining the effect of chloroform on the muscle membrane potential, a series of solutions of varied concentration of chloroform were used. Only slight hyperpolarization was observed with 0.1% chloroform in saline. At 0.2%, a notable rapid hyperpolarization was observed (Figure 2). The muscle would sometimes spontaneously show a wave of hyperpolarization with exposure to 0.2%. A 0.3% concentration would also produce a rapid hyperpolarization as would a 1% exposure. If an initial exposure of 0.3% or higher is used and left on the preparation, the muscle would start to produce waves of contraction. At 1% or higher the contraction would be so great as to cause the cuticle to tear where it was pinned. It was difficult to maintain an intracellular recording in such conditions (Figure 2C). The muscles would then stay in a state of contraction, and when they would relax, they appeared damaged and were not translucent as observed in healthy, freshly dissected preparations. Thus, subsequent use of chloroform was at 0.2% in order to obtain recordings long enough before the muscle would contract and remain in the state of contraction. However, a 0.2% concentration still provided a substantial hyperpolarization of the muscle in every preparation examined (*N* = 6, *p* < 0.05, Paired *t*-test).

### 2.2. Effect LPS on the Resting Membrane Potential and LPS Combined with Chloroform

**Figure 3:**
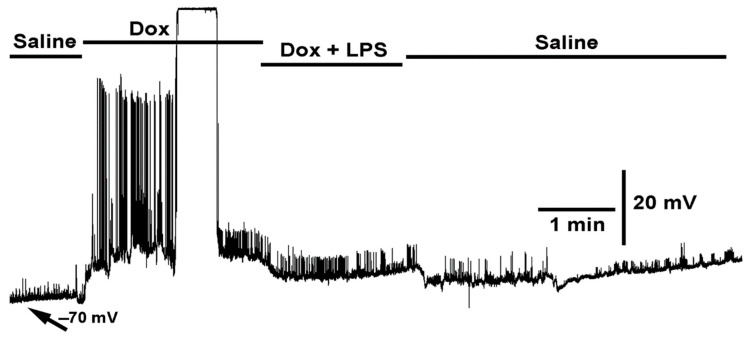
The effect of doxapram (10 mM) and the cocktail of doxapram (10 mM) and LPS on membrane potential and synaptic transmission. Exposure to doxapram (10 mM) rapidly depolarizes the membrane potential and evoked responses, from transected segmental nerves from the CNS. The motor neurons depolarize producing excitatory junction potentials (EJPs) of similar amplitude as those of electrical evoked nerve EJPs. The barraged of depolarizations dislodged the intracellular recordings. The bath exchange to doxapram and LPS produced small hyperpolarization while small EJPs and mEJPs are still present. Upon exchanging to fresh saline, free of doxapram and LPS, the membrane potential almost regained the initial resting membrane potential.

Before testing the effects of LPS (250 µg/mL) with chloroform (0.2%) on membrane potential, the effect of (1) doxapram (10 mM), (2) doxapram with LPS (250 µg/mL), and (3) doxapram with chloroform were first explored. Exposure of the muscle to doxapram at 10 mM rapidly produced a depolarization and induced evoked-like EJPs (Figure 3). The EJPs likely occurred due to depolarization of the motor neuron terminals still attached to the muscle fibers. The motor neurons were transected from the CNS but remained intact to the muscle fibers. Upon addition of saline of doxapram combined with LPS, the rapid hyperpolarization normally induced by LPS was blocked. It is also important to note that the small EJPs, likely evoked by the motor neuron and the spontaneous quantal events (i.e., mEJPs), still occur in the presence of doxapram combined with LPS. This indicates that LPS is not fully blocking the glutamate receptors if doxapram is applied first.

**Figure 4:**
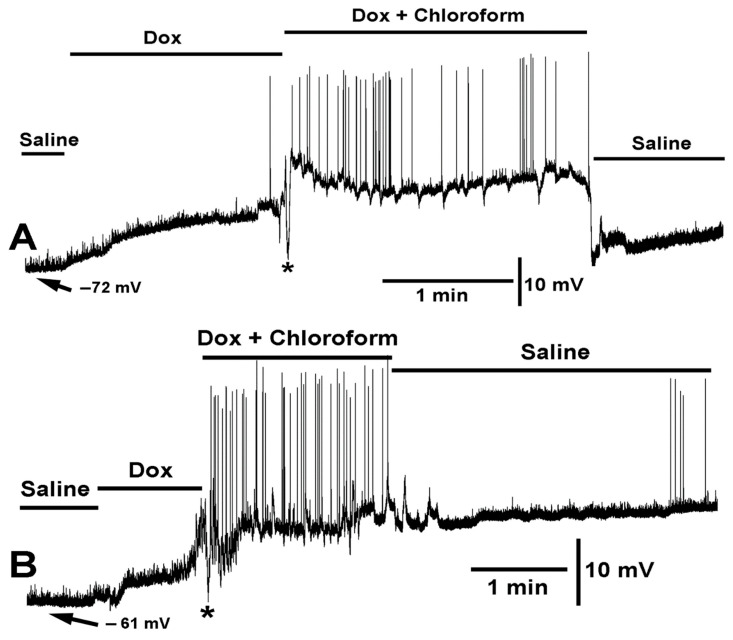
Two variations in the effect of doxapram (10 mM) and the cocktail of doxapram (10 mM) and chloroform (0.2%) are shown. (**A**,**B**) illustrate two different preparations of the same concentrations of compounds and mixtures. In both variations, doxapram depolarized the membrane potential and upon switching to the cocktail of doxapram (10 mM) and chloroform (0.2%), the membrane rapidly hyperpolarized (<1 s, see asterisks *) followed with depolarization and doxapram induced EJPs. Exchange to fresh saline recovered the membrane potential in (**A**) but doxapram had a lingering effect in some preparations as shown in (**B**).

When doxapram (10 mM) was first applied, resulting in a depolarized state, and then chloroform (0.2%) applied with doxapram (10 mM), a rapid but very transient hyperpolarizing response occurred. To illustrate the variation in responses across preparations of the same concentrations of the compounds and cocktail mixtures, two different preparations are highlighted (Figure 4A,B). As shown in Figure 4A, the muscle started to show evoked like EJPs toward the end of the first exposure to doxapram (10 mM) and continued throughout the exposure to the cocktail mixture. Most relevant is to note that chloroform still produced hyperpolarization responses in the presence of doxapram as indicated with the asterisks in both Figure 4A,B. Another known antagonist to K2P channels, PK-THPP, [57] was examined at 1 mM and 10 mM. There was no effect on the resting membrane potential over 2 min of exposure, and no alterations in the frequency or amplitude of spontaneous quantal events (*N* = 6, *p* > 0.05, paired *t*-test).

**Figure 5:**
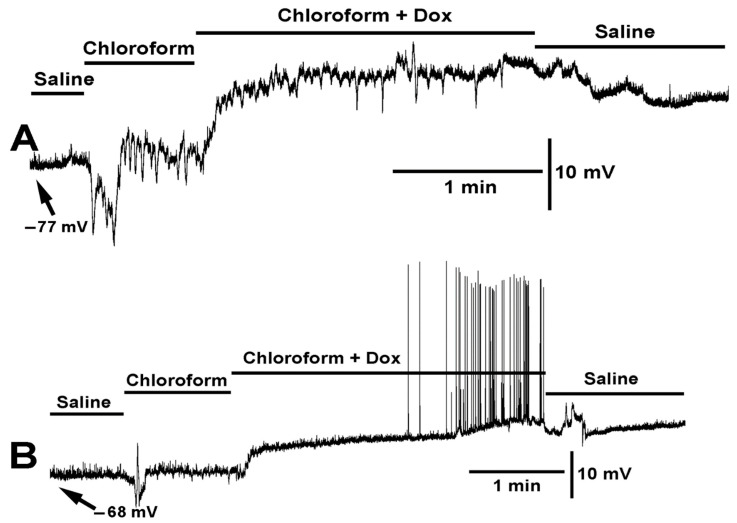
Two examples of the effect on membrane potential by chloroform (0.2%) and chloroform (0.2%) combined with doxapram (10 mM) are shown (**A**,**B**). Two different preparations are shown with the same mixtures of compounds. Upon exposure to chloroform the membrane rapidly hyperpolarized followed by depolarization. In (**A**), chloroform produced a series of contractions. With the exposure to chloroform and doxapram the membrane depolarized even more. In (**B**), doxapram induced EJPs, while in A the effect of chloroform continues to produce waves of contraction.

When the treatment sequence was changed to apply chloroform (0.2%) first, followed by a cocktail of chloroform (0.2%) and doxapram (10 mM), a different effect was observed. Chloroform (0.2%) produced the initial hyperpolarization followed by small to large waves of muscle contraction upon exposure. When a cocktail of chloroform (0.2%) and doxapram (10 mM) was applied, the muscle depolarized rapidly (*N* = 6, *p* < 0.05, paired *t*-test). To highlight the variation in responses observed across trials with different preparations of chloroform (0.2%) followed by a cocktail of chloroform (0.2%) and doxapram (10 mM), two different preparations are shown (Figure 5A,B). In both examples, the initial chloroform induced hyperpolarization is shown. Additionally, in both examples, doxapram rapidly produced depolarization. However, in Figure 5B the Doxapram induced evoked EJPs in the presence of chloroform. In Figure 5A, the multiple stints of hyperpolarization due to chloroform continued to occur while exposed also to doxapram. This response was similar in the paradigm when doxapram was applied first followed by chloroform combined with doxapram (Figure 4B).

**Figure 6:**
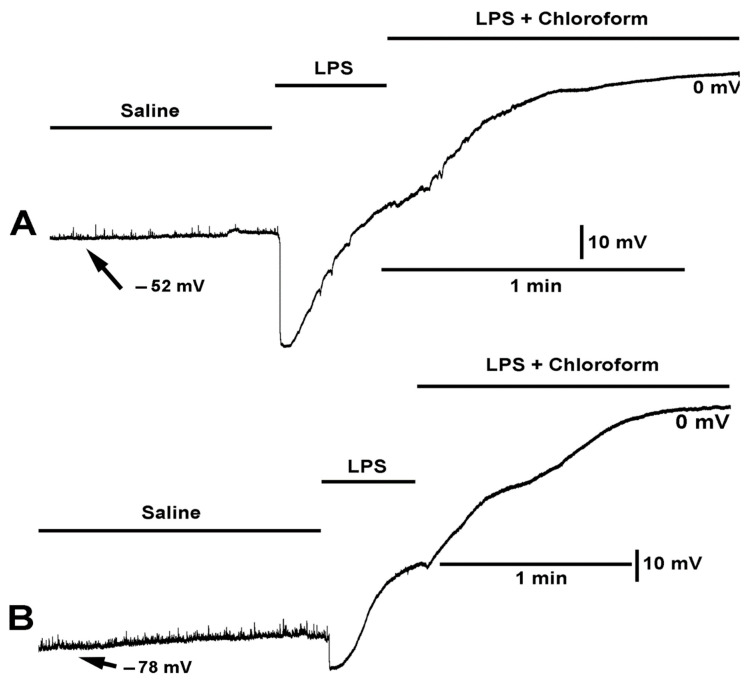
The effect on the resting membrane potential by LPS and LPS combined with chloroform (0.2%). (**A**,**B**) Two different preparations are shown with the same mixtures of compounds. LPS produced a larger hyperpolarization in preparation shown in (**A**) than in the preparation shown in (**B**). Upon exposure of LPS combined with chloroform the membrane potential continued to depolarize to zero.

To examine if chloroform would still be able to hyperpolarize the membrane in the presence of LPS, LPS was applied first followed by a cocktail of LPS and chloroform (0.2%). The initial LPS exposure produced the typical hyperpolarization but upon applying the cocktail of LPS and chloroform the membrane did not show a rapid hyperpolarization but continued to depolarize (*N* = 6, *p* < 0.05, paired *t*-test; Figure 6). To highlight the variation in responses observed, two different preparations are shown (Figure 6A,B). In both preparations, example traces show the same trends. The slight pause in depolarization may be an indication that chloroform in the cocktail mixture has some action but very minor in comparison to chloroform on its own (Figure 5). During the combined exposure with LPS, it did not appear that chloroform was producing waves of muscle contraction. It was as if LPS inhibited the action of chloroform.

**Figure 7:**
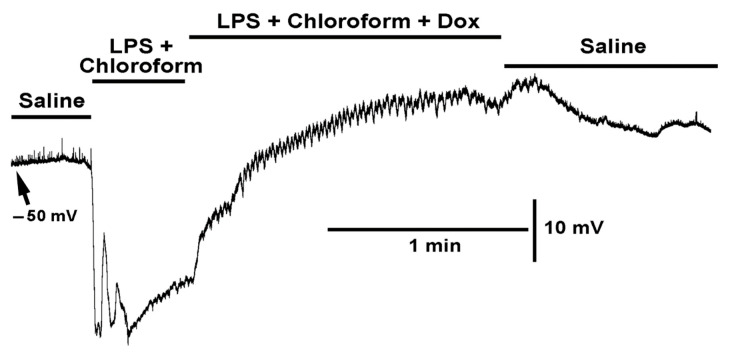
The effect on the resting membrane potential with exposure to LPS combined with chloroform (0.2%) and LPS combined with both chloroform (0.2%) and doxapram (10 mM). The combined LPS with chloroform still produced rapid hyperpolarization, but with some rapid depolarizations which is unlike LPS exposure by itself. LPS combined with both chloroform and doxapram rapidly depolarized the membrane and resulted in waves on contraction. The effects of the cocktail can partially be reversed by saline wash.

When LPS and chloroform were initially applied together, a rapid hyperpolarization would occur along with small waves of muscle contraction (Figure 7). Thus, the muscle was able to produce contractions while being hyperpolarized. The additional exposure of doxapram along with the cocktail of LPS and chloroform still produced a rapid depolarization. Thus, the presence of LPS and chloroform did not block the action of doxapram.

### 2.3. Effect of Electrically Stimulated Nerve While Exposed to Various Preparations

**Figure 8:**
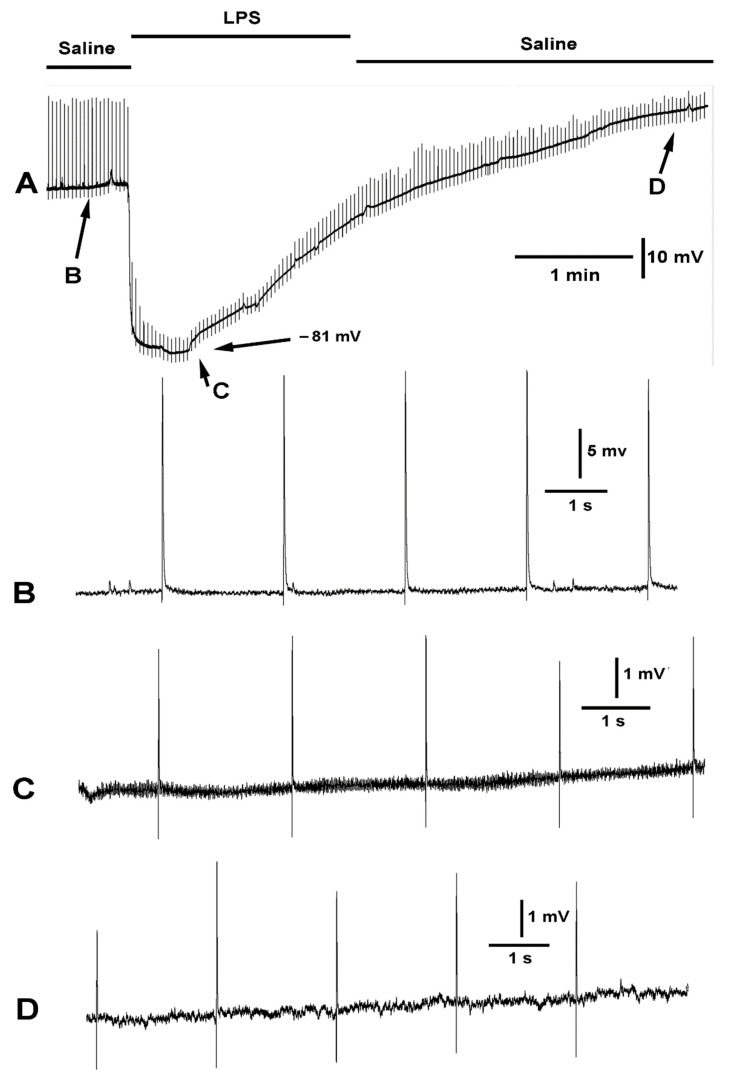
The effect of LPS on evoked synaptic transmission and resting membrane potential. The segmental nerve was stimulated at a rate of 0.5 Hz. (**A**) The overall effect on the membrane potential and the amplitude of the EJPs is shown. Note the membrane potential started to repolarize while still exposed to LPS. (**B**) The EJPs and mEJPs were observed in saline. (**C**) Exposure to LPS rapidly hyperpolarized the membrane and reduced the amplitude of the evoked EJPs and the mEJPs. The mEJPs can no longer be observed. Despite the increased driving force for increased amplitude of the EJPs and mEJPs they were greatly reduced in amplitude. (**D**) When the bath was exchanged to fresh saline, the membrane regains the initial value and the amplitude of the evoked EJPs increased.

As indicated previously, LPS does hyperpolarize the muscle, but it also blocks the small depolarizations of mEJPs as well as the nerve evoked EJPs (Figure 8). Considering the observed effects of the compounds presented above (doxapram, chloroform, LPS) specifically acting on the membrane potential of the muscle, and the subsequent effect likely leading to the motor neurons being able to depolarize to produce the evoked like EJPs as shown during exposure with doxapram (Figure 3, Figure 4 and Figure 5), the segmental nerve was electrically stimulated while exposing the preparation to these same compounds to examine the effects.

**Figure 9:**
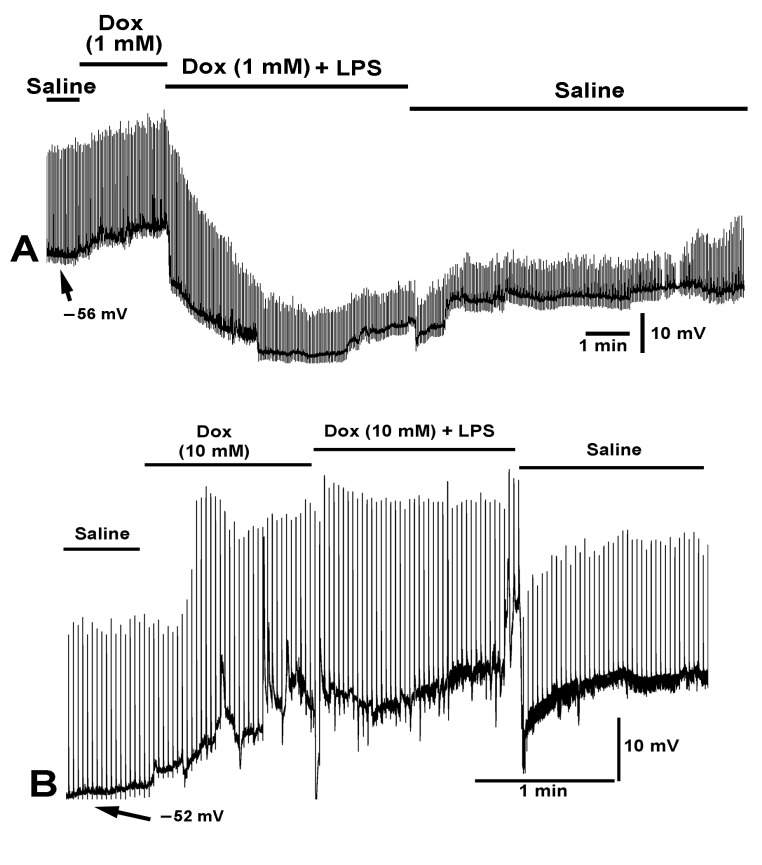
The effect of doxapram (1 and 10 mM) and a cocktail of doxapram (1 and 10 mM) with LPS on membrane potential and evoked EJPs. The segmental nerve was stimulated at a rate of 0.5 Hz. (**A**,**B**) Two different preparations are shown with varied concentrations of doxapram. (**A**) A concentration of 1 mM doxapram resulted in depolarization of the membrane and the combination of doxapram (1 mM) with the same concentration (250 µg/mL) of LPS as used in B produced a hyperpolarization without the rapid depression in the amplitudes of the evoked EJPs. Upon exchanging the media for fresh saline, the amplitudes of the EJPs increased and the membrane potential trended towards regaining the initial value. (**B**) Doxapram at 10 mM produced a large depolarization and increased the amplitude of the evoked EJPs despite a reduced driving gradient due to the membrane being depolarized. The subsequent exchange of the media to doxapram (10 mM) and LPS (250 µg/mL) did not maintain a hyperpolarized state and the evoked as well as mEJPs were not greatly affected by the LPS. Upon exchanging the bath to fresh saline, the evoked EJPs reduced in amplitude but a high frequency of mEJPs remained.

In some cases, doxapram produced a slight increase in the amplitude of the EJPs (Figure 9A), while in other cases larger increases were observed (Figure 9B) even while the membrane potential of the muscle was undergoing depolarization. Given that the driving gradient for the evoked EJPs would generally decrease as the muscle depolarizes, the cause of the large EJPs by doxapram is likely due to enhanced vesicle fusion events from the motor nerve terminal depolarizing. A most intriguing finding was that if preparations are first exposed to a low concentration of doxapram (1 mM) and then LPS, the glutamate receptors appear not to be as severely blocked by LPS since the EJPs are still present and the membrane still hyperbolized (Figure 9A). However, if a higher concentration of doxapram was first used (10 mM) and then the same concentration of LPS was applied, the effects of LPS were blocked in both hyperpolarizing the membrane as well as blocking the glutamate response (Figure 9B).

**Figure 10:**
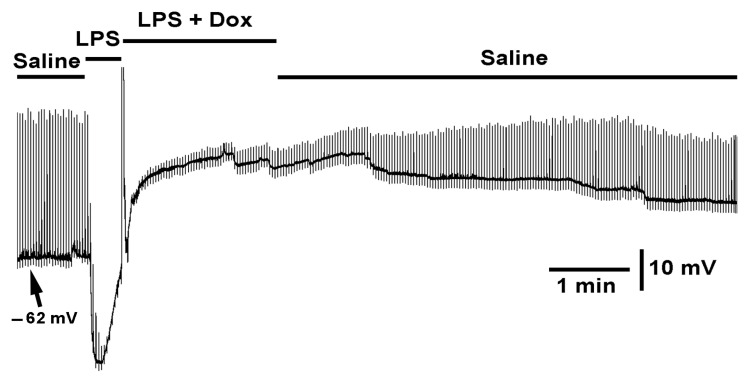
The effect of LPS and the cocktail of LPS with doxapram on membrane potential and evoked EJPs. The segmental nerve was stimulated at a rate of 0.5 Hz. LPS rapidly hyperpolarized the membrane and depressed evoked synaptic transmission. The bath exchange to LPS and doxapram (10 mM) rapidly depolarized the membrane and does not fully relive the action of LPS in blocking synaptic transmission but allowed the synaptic response to increase in amplitude.

When LPS was initially applied followed by doxapram with LPS, only the hyperpolarization response by LPS was rapidly reversed with a slight increase in responsiveness to the evoked glutamate reappearing (Figure 10, *N* = 6, *p* < 0.05, paired *t*-test).

## 3. Discussion

Previous to this study there was no indication that LPS acted on K2P channels. Additionally, no known blocker has yet been identified to stop the direct action of LPS in hyperpolarizing the membrane or inhibiting the action on glutamate receptors. Only studies from our research group have reported on the hyperpolarizing effects of LPS on skeletal muscle [56]. Our focus has been on the *Drosophila* model due to the suggestions that the known receptors for LPS were PGRP-LC and PGRP-LE. The direct actions responsible for the rapid effect by LPS in membrane hyperpolarization and blocking the response to glutamate are not mediated by PGRP-LC and PGRP-LE [17]. In addition, there are no known reports that doxapram, a K2P channel blocker, would retard the responses induced by LPS. These preliminary findings, reported herein, are significant and are the tip of the iceberg as much remains to be investigated. For example, full dose–response curves for each interaction are needed for the skeletal muscle as well as in other cell types, such as neurons. In addition, there are many strains of Gram-negative bacteria, each with a unique form of LPS. To screen all the LPS forms and concentrations for physiological effects will consume years of research. It is our hope that presenting this overview in *Drosophila* as a model organism initiates future studies that will prevail and potentially lead to new therapeutics in blocking the responses to LPS since it is a major health concern for humans and in veterinary care in cases of bacterial induced septicemia.

The rapid action of LPS inducing hyperpolarization of the larval muscle suggests that the membrane potential is being driven to the estimated equilibrium potential for K^+^ (E_K_). It was reported earlier that the adult *Drosophila* muscle and moth muscle have an equilibrium potential for K^+^ more negative than −90 mV [58,59]. Thus, if the K2P channels are activated by LPS, then either the K2P channel is showing inactivation, or the conformation is not allowing LPS to remain bound since the effect is transitory but longer in duration than the action for chloroform. In speculation, the LPS could activate the K2P channel and then block it, as the membrane continues to depolarize above the resting membrane potential if muscle remains exposed to LPS. Other possibilities such as hyperactivation of the Na-K ATPase pump or a Cl^-^ ion influx or even activation of a calcium activated potassium channel have been tested earlier and showed not to contribute to the action by LPS [18,47,60].

The type of K2P channel expressed in larval *Drosophila* muscle is not yet known. It is likely the Tandem-Pore Weak Inward rectifying K^+^ channel (TWIK-1) and/or the TASK-1 and TASK-3 (Tandem-pore Acid-Sensing K+ channels) as these subtypes are known to be sensitive to low pH as is found in the larval *Drosophila* and crayfish muscle which depolarize in lower pH [46,56,61,62]. Since the TASK-1 is activated only halothane and isoflurane [44] and there is some hyperpolarization induced with chloroform with the *Drosophila* muscle, it would suggest a TASK-1 like K2P channel is present. There may be various K2P channels subtypes expressed in a given cell, but this has yet to be investigated. The research around K2P channels subtypes and pharmacological profiles is growing [22,36,37,38,39,40,41] but there is still much to be learned about these channels particularly related to disease states [25].

The rationale to use the LPS form *Serratia marcescens* for these studies was to follow through with earlier studies using larval *Drosophila* which reported on *S. marcescens* to alter heart rate [63] and CNS actions [64] as well as *S. marcescens* still producing large effects in RNAi lines for both receptor complexes PGRP-LC and PGRP-LE [17]. In addition, repetitive action in exposure to varied concentrations of LPS from *S. marcescens* was performed [65]. It was also demonstrated that muscle in crayfish hyperpolarized due to exposure of LPS from *S. marcescens* [66]. Other forms of LPS may be even more or less potent in their effects in comparison to what has been reported for *S. marcescens*. However, if actions of other forms also work through activation of K2P channels and can be preemptively blocked by doxapram, then a commonality in the action of LPS may apply to various other organisms as well [67]. Much lower concentrations of doxapram (0.41 to 37 μM), than used in this study, have significant effects in mammalian preparations [31] which may indicate a different K2P receptor subtype with a lower affinity on the larval muscle than neurons of mammals.

Screening for more potent K2P blockers would be of interest and could help in mediating a LPS response. For example, verapamil was recently investigated as it has been established to block the TRESK subtype of K2P channels [68]. In addition, PK-THPP has been found to mimic the effects of doxapram in carotid body type-1 or glomus cells with patch clamp techniques [57] and be even more sensitive to increase breathing rate in rodents than doxapram [69]. The compound ML365 was also shown to have similar effects as doxapram and PK-THPP [57]. However, PK-THPP had no effect on the larval *Drosophila* muscle even at a high concentration of 10 mM. As more specific pharmacological compounds are examined for K2P channels and comparisons with compounds like doxapram and verapamil [68], the subtypes will be better characterized in various organisms and cell types [47,70]. Perhaps the muscle in the larval *Drosophila* can aid in serving as a model for this purpose as well.

## 4. Materials and Methods

The same procedures and larval *Drosophila* were used as described in Vacassenno et al., [18,47] and highlighted below. The few exceptions in the protocols reported herein include the use of different compounds, or the combination of combined compounds and their varied concentrations.

### 4.1. Animals

*Drosophila melanogaster*, Canton S (CS) flies were used in all physiological assays. This strain has been isogenic in the lab for several years and was originally obtained from Bloomington Drosophila Stock Center (BDSC). All animals were maintained in vials partially filled with a cornmeal-agar-dextrose-yeast medium.

### 4.2. Neuromuscular Junctions of Larval Drosophila

Early third instar larval *D. melanogaster* were dissected in physiological saline. To monitor the transmembrane potentials of the body wall muscle (m6) of 3rd instar larvae, a sharp intracellular electrode (30 to 40 M resistance) filled with 3M KCl impaled the fiber. An Axoclamp 2B (Molecular Devices, Sunnyvale, CA, USA) amplifier and 1 X LU head stage was used. The preparations were dissected in physiological saline [71,72]: (NaCl 70 mM, KCl 5 mM, MgCl_2_·6H_2_O 20 mM, NaHCO_3_ 10 mM, Trehalose 5 mM, sucrose 115 mM, BES 25 mM, and CaCl_2_·2H_2_O 1 mM, pH 7.1). All experiments were performed at room temperature (20–21 °C).

### 4.3. Chemicals

Doxapram or PK-THPP powder was added directly to the saline to obtain 10 mM and placed on a vortex (high setting) for 5 min. The saline solution remains opaque. Dilutions from these stocks were used at various other concentrations as indicated in the Results. LPS from *Serratia marcescens* was dissolved in physiological saline the day of experimentation. A high concentration of LPS (250 µg/mL) was used to compare with previous studies (250 µg/mL) to best address the mechanisms of action [56]. The lethal dose for 50% of survival (LD50) in rodents for LPS from *S. marcescens* is 650 µg/mL (i.e., 6 × 10^6^ CFU colony-forming units) [73]. This was the justification to use a relatively high concentration for *Drosophila* since they are likely exposed to high levels of Gram-negative bacterial strains in their native environment. Chemicals were obtained from Sigma-Aldrich (St. Louis, MO, USA), except PK-THPP was obtained from Aberjona Laboratories (Boston, MA, USA).

Chloroform was spectrophotometric grade (99.9% pure; FW = 119.4 g/mole). It was determined in this study that 13.6 mM was sufficient to provide a response without immediately contracting the muscle and dislodging the intracellular electrode. This concentration was obtained by using 100 mL of the stock and mixing it with 5 mL of physiological saline. The solution was vortexed and then 50 mL of this solution was used to place into the 5 mL of the solution to be exposed to the preparation. Solutions were made fresh within 2 h of use and were stored in airtight vials between uses. Solutions were shaken prior to being exposed to the preparation. Note: Chloroform would dissolve the magnetic strips so multiple recording dishes were needed and replaced when using the high concentrations. The final concentration used (13.6 mM) was not as noticeable in dissolving the magnetic strips and was not as noticed by inhalation around the recording rig. However, care still needed to be taken not to inhale the vapors while placing the microelectrodes for recordings.

The exchanging of the media did result in small deflections in the recordings in some cases, but these are usually only during the exchange and can be noted easily in the recording as artifactual (see [18]). However, with the use of an agar bridge for the ground in the bath the electrical deflections from changing the media are kept to a minimum. The 1% agar plug was constructed within a 200 µL Eppendorf pipette tip, made with normal saline. The plastic pipette tip prevents a DC offset from varying saline levels on the ground lead within the recording dish when changing the media. The tip of the pipette stays in an electrical contact with the preparation during the bath exchanges.

### 4.4. Statistical Methods

In general, when normality was to be assumed, Shapiro–Wilks tests were used to validate the assumptions in order to determine the use of a *t*-test or a Wilcoxon Signed Rank Test. A significant difference is considered as *p* < 0.05.

## Data Availability

The data are presented in the manuscript and available on request.

## References

[1] Osborn M.J., Rosen S.M., Rothfield L., Zeleznick L.D., Horecker B.L. (1964). Lipopolysaccharide of the gram-negative cell wall. Science.

[2] Linhartová I., Bumba L., Mašín J., Basler M., Osička R., Kamanová J., Prochazkova K., Adkins I., Holubova J., Sadilkova L. (2010). RTX proteins: A highly diverse family secreted by a common mechanism. FEMS Microbiol. Rev..

[3] Da Silva Correia J., Soldau K., Christen U., Tobias P.S., Ulevitch R.J. (2001). Lipopolysaccharide is in close proximity to each of the proteins in its membrane receptor complex. transfer from CD14 to TLR4 and MD-2. J. Biol. Chem..

[4] Wilson J.X., Young G.B. (2003). Progress in clinical neurosciences: Sepsis-associated encephalopathy: Evolving concepts. Can. J. Neurol. Sci..

[5] Friedrich O., Reid M.B., Van den Berghe G., Vanhorebeek I., Hermans G., Rich M.M., Larsson L. (2015). The sick and the weak: Neuropathies/myopathies in the critically ill. Physiol. Rev..

[6] Al-Nassan S., Fujino H. (2018). Exercise preconditioning attenuates atrophic mediators and preserves muscle mass in acute sepsis. Gen. Physiol. Biophys..

[7] Eidelman L.A., Putterman D., Putterman C., Sprung C.L. (1996). The spectrum of septic encephalopathy definitions, etiologies, and mortalities. JAMA.

[8] Levin T.C., Malik H.S. (2017). Rapidly evolving Toll-3/4 genes encode male-specific Toll-like receptors in Drosophila. Mol. Biol. Evol..

[9] Coscia M., Giacomelli S., Oreste U. (2011). Toll-like receptors: An overview from invertebrates to vertebrates. Invert. Surv. J..

[10] Loker E.S., Adema C.M., Zhang S.M., Kepler T.B. (2004). Invertebrate immune systems—Not homogeneous, not simple, not well understood. Immunol. Rev..

[11] Kleino A., Silverman N. (2014). The Drosophila IMD pathway in the activation of the humoral immune response. Dev. Com. Immunol..

[12] Gottar M., Gobert V., Michel T., Belvin M., Duyk G., Hoffmann J.A., Ferrandon D., Royet J. (2002). The Drosophila immune response against Gram-negative bacteria is mediated by a peptidoglycan recognition protein. Nature.

[13] Takehana A., Katsuyama T., Yano T., Oshima Y., Takada H., Aigaki T., Kurata S. (2002). Overexpression of a pattern-recognition receptor, peptidoglycan recognition protein-LE, activates imd/relish mediated antibacterial defense and the prophenoloxidase cascade in Drosophila larvae. Proc. Natl. Acad. Sci. USA.

[14] Leclerc V., Reichhart J.M. (2004). The immune response of Drosophila melanogaster. Immunol. Rev..

[15] Werner T., Liu G., Kang D., Ekengren S., Steiner H., Hultmark D. (2000). A family of peptidoglycan recognition proteins in the fruit fly *Drosophila melanogaster*. Proc. Natl. Acad. Sci. USA.

[16] Perkins L.A., Holderbaum L., Tao R., Hu Y., Sopko R., McCall K., Yang-Zhou D., Flockhart I., Binari R., Shim H.-S. (2015). The transgenic RNAi project at Harvard Medical School: Resources and validation. Genetics.

[17] Ballinger-Boone C., Anyagaligbo O., Bernard J., Bierbower S.M., Dupont-Versteegden E.E., Ghoweri A., Greenhalgh A., Harrison D., Istas O., McNabb M. (2020). The effects of bacterial endotoxin (LPS) on cardiac and synaptic function in various animal models: Larval Drosophila, crayfish, crab, and rodent. Internat. J. Zool. Res..

[18] Vacassenno R.M., Haddad C.N., Cooper R.L. (2022). Lipopolysaccharide (LPS) action on hyperpolarizing membrane potential: Antagonized by the K2P channel blocker, Doxapram, and independent of calcium activated potassium channels.

[19] Ueda I., Hirakawa M., Arakawa K., Kamaya H. (1986). Do anesthetics fluidize membranes?. Anesthesiology.

[20] Hao X., Ou M., Zhang D., Zhao W., Yang Y., Liu J., Yang H., Zhu T., Li Y., Zhou C. (2020). The effects of general anesthetics on synaptic transmission. Curr. Neuropharmacol..

[21] Buckingham S.D., Kidd J.F., Law R.J., Franks C.J., Sattelle D.B. (2005). Structure and function of two-pore-domain K+ channels: Contributions from genetic model organisms. Trends Pharmacol. Sci..

[22] Plant L.D., Goldstein S.A.N., Zheng J., Trudeau M.C. (2015). Two-Pore Domain Potassium Channels. Handbook of Ion Channels.

[23] Goldstein S.A., Price L.A., Rosenthal D.N., Pausch M.H. (1996). ORK1, a potassium-selective leak channel with two pore domains cloned from Drosophila melanogaster by expression in Saccharomyces cerevisiae. Proc. Natl. Acad. Sci. USA.

[24] Goldstein S.A., Wang K.W., Ilan N., Pausch M.H. (1998). Sequence and function of the two P domain potassium channels: Implications of an emerging superfamily. J. Mol. Med..

[25] Lee L.M., Müntefering T., Budde T., Meuth S.G., Ruck T. (2021). Pathophysiological role of K2P channels in human diseases. Cell Physiol. Biochem..

[26] Schmidt C., Wiedmann F., Schweizer P.A., Katus H.A., Thomas D. (2014). Inhibition of cardiac two-pore-domain K+ (K2P) channels--an emerging antiarrhythmic concept. Eur. J. Pharmacol..

[27] Cotten J.F., Keshavaprasad B., Laster M.J., Eger E.I., Yost C.S. (2006). The ventilatory stimulant doxapram inhibits TASK tandem pore (K2P) potassium channel function but does not affect minimum alveolar anesthetic concentration. Anesth. Analg..

[28] Komatsu R., Sengupta P., Cherynak G., Wadhwa A., Sessler D.I., Liu J., Hurst H.E., Lenhardt R. (2005). Doxapram only slightly reduces the shivering threshold in healthy volunteers. Anesth. Analg..

[29] Yost C.S. (2006). A new look at the respiratory stimulant doxapram. CNS Drug Rev. Fall-Winter.

[30] Song S.S., Lyden P.D. (2012). Overview of therapeutic hypothermia. Curr. Treat. Options. Neurol..

[31] Cunningham K.P., MacIntyre D.E., Mathie A., Veale E.L. (2020). Effects of the ventilatory stimulant, doxapram on human TASK-3 (KCNK9, K2P9.1) channels and TASK-1 (KCNK3, K2P3.1) channels. Acta Physiol..

[32] Vliegenthart R.J., Ten Hove C.H., Onland W., van Kaam A.H. (2017). Doxapram treatment for apnea of prematurity: A systematic review. Neonatology.

[33] Sauter C., Wolfensberger C. (1980). Interferon in human serum after injection of endotoxin. Lancet.

[34] Adams M.D., Celniker S.E., Holt R.A., Evans C.A., Gocayne J.D., Amanatides P.G., Scherer S.E., Li P., Hoskins R.A., Galle R. (2000). The genome sequence of Drosophila melanogaster. Science.

[35] Littleton J.T., Ganetzky B. (2000). Ion channels and synaptic organization: Analysis of the Drosophila genome. Neuron.

[36] Enyedi P., Braun G., Czirják G. (2012). TRESK: The lone ranger of two-pore domain potassium channels. Mol. Cell Endocrinol..

[37] Kamuene J.M., Xu Y., Plant L.D. (2021). The pharmacology of two-pore domain potassium channels. Handb. Exp. Pharmacol..

[38] Duprat F., Lesage F., Fink M., Reyes R., Heurteaux C., Lazdunski M. (1997). TASK, a human background K+ channel to sense external pH variations near physiological pH. EMBO J..

[39] Kim Y., Bang H., Kim D. (2000). TASK-3, a new member of the tandem pore K(+) channel family. J. Biol. Chem..

[40] Rajan S., Wischmeyer E., Xin Liu G., Preisig-Muller R., Daut J., Karschin A., Derst C. (2000). TASK-3, a novel tandem pore domain acid-sensitive K+ channel. An extracellular histidine as pH sensor. J. Biol. Chem..

[41] Kim D. (2005). Physiology and pharmacology of two-pore domain potassium channels. Curr. Pharm. Des..

[42] Mu D., Chen L., Zhang X., See L.-H., Koch C.M., Yen C., Tong J.J., Spiegel L., Nguyen K.C., Servoss A. (2003). Genomic amplification and oncogenic properties of the KCNK9 potassium channel gene. Cancer Cell.

[43] Holter J., Carter D., Leresche N., Crunelli V., Vincent P. (2005). A TASK3 channel (KCNK9) mutation in a genetic model of absence epilepsy. J. Mol. Neurosci..

[44] Patel A.J., Honore E., Lesage F., Fink M., Romey G., Lazdunski M. (1999). Inhalational anesthetics activate two-pore-domain background K channels. Nat. Neurosci..

[45] Tian F., Qiu Y., Lan X., Li M., Yang H., Gao Z. (2019). A small-molecule compound selectively activates K2P channel TASK-3 by acting at two distant clusters of residues. Mol. Pharmacol..

[46] Badre N.H., Martin M.E., Cooper R.L. (2005). The physiological and behavioral effects of carbon dioxide on Drosophila melanogaster larvae. Comp. Biochem. Physiol. A..

[47] Vacassenno R.M., Haddad C.N., Cooper R.L. (2023). The effects on resting membrane potential and synaptic transmission by Doxapram (blocker of K2P channels) at the *Drosophila* neuromuscular junction. Comp. Biochem. Physiol. C.

[48] Kollert S., Döring F., Gergs U., Wischmeyer E. (2020). Chloroform is a potent activator of cardiac and neuronal Kir3 channels. Naunyn Schmiedebergs Arch. Pharmacol..

[49] Andres-Enguix I., Caley A., Yustos R., Schumacher M.A., Spanu P.D., Dickinson R., Maze M., Franks N.P. (2007). Determinants of the anesthetic sensitivity of two-pore domain acid-sensitive potassium channels: Molecular cloning of an anesthetic-activated potassium channel from *Lymnaea stagnalis*. J. Biol. Chem..

[50] Yamaguchi M., Yoshida H. (2018). Drosophila as a model organism. Adv. Exp. Med. Biol..

[51] Ugur B., Chen K., Bellen H.J. (2016). Drosophila tools and assays for the study of human diseases. Dis. Model. Mech..

[52] Ecovoiu A.A., Ratiu A.C., Micheu M.M., Chifiriuc M.C. (2022). Inter-Species Rescue of Mutant Phenotype-The Standard for Genetic Analysis of Human Genetic Disorders in *Drosophila melanogaster* Model. Int. J. Mol. Sci..

[53] Dow J.A.T., Simons M., Romero M.F. (2022). Drosophila melanogaster: A simple genetic model of kidney structure, function and disease. Nat. Rev. Nephrol..

[54] Shin G.J., Abaci H.E., Smith M.C. (2022). Cellular pathogenesis of chemotherapy-induced peripheral neuropathy: Insights from *Drosophila* and Human-engineered skin models. Front. Pain Res..

[55] Mackay T.F., Anholt R.R. (2006). Of flies and man: Drosophila as a model for human complex traits. Annu. Rev. Genom. Hum. Genet..

[56] Cooper R.L., McNabb M., Nadolski J. (2019). The effects of a bacterial endotoxin LPS on synaptic transmission at the neuromuscular junction. Heliyon.

[57] O’Donohoe P.B., Huskens N., Turner P.J., Pandit J.J., Buckler K.J. (2018). A1899, PK-THPP, ML365, and Doxapram inhibit endogenous TASK channels and excite calcium signaling in carotid body type-1 cells. Physiol. Rep..

[58] Ikeda K., Ozawa S., Hagiwara S. (1976). Synaptic transmission reversibly conditioned by single-gene mutation in Drosophila melanogaster. Nature.

[59] Salkoff L.B., Wyman R.J. (1983). Ion currents in Drosophila flight muscles. J. Physiol..

[60] Potter R., Meade A., Potter S., Cooper R.L. (2021). Rapid and direct action of lipopolysaccharides (LPS) on skeletal muscle of larval Drosophila. Biology.

[61] Bierbower S.M., Cooper R.L. (2010). The effects of acute carbon dioxide on behavior and physiology in *Procambarus clarkii*. J. Exp. Zool..

[62] Bierbower S.M., Shuranova Z.P., Viele K., Cooper R.L. (2013). Comparative study of environmental factors influencing motor task learning and memory retention in sighted and blind crayfish. Brain Behav..

[63] Anyagaligbo O., Bernard J., Greenhalgh A., Cooper R.L. (2019). The effects of bacterial endotoxin (LPS) on cardiac function in a medicinal blow fly (*Phaenicia sericata*) and a fruit fly (*Drosophila melanogaster)*. Comp. Biochem. Physiol. C.

[64] Istas O., Greenhalgh A., Cooper R.L. (2019). The effects of a bacterial endotoxin on behavior and sensory-CNS-motor circuits in *Drosophila melanogaster*. Insects.

[65] Istas O., Greenhalgh A., Cooper R.L. (2020). Repetitive exposure to bacterial endotoxin LPS alters synaptic transmission. J. Pharmacol. Toxicol..

[66] Saelinger C.M., McNabb M.C., McNair R., Bierbower S., Cooper R.L. (2019). Effects of bacterial endotoxin (LPS) on the cardiac function, neuromuscular transmission and sensory-CNS-motor nerve circuit: A crustacean model. Comp. Biochem. Physiol. A.

[67] Keshavaprasad B., Liu C., Au J.D., Kindler C.H., Cotten J.F., Yost C.S. (2005). Species-specific differences in response to anesthetics and other modulators by the K2P channel TRESK. Anesth. Analg..

[68] Park H., Kim E.-J., Ryu J.H., Lee D.K., Hong S.-G., Han J., Han J., Kang D. (2018). Verapamil inhibits TRESK (K2P18.1) current in trigeminal ganglion neurons independently of the blockade of Ca^2+^ influx. Int. J. Mol. Sci..

[69] Cotton J.F. (2013). TASK-1 (KCNK3) and TASK-3 (KCNK9) tandem pore potassium channel antagonists stimulate breathing in isoflurane-anesthetized rats. Anesth. Analg..

[70] Ison B.J., Abul-Khoudoud M.O., Ahmed S., Alhamdani A.W., Ashley C., Bidros P.C., Bledsoe C.O., Bolton K.E., Capili J.G., Henning J.N. (2022). The effect of doxapram on proprioceptive neurons: Invertebrate model. NeuroSci.

[71] Stewart B.A., Atwood H.L., Renger J.J., Wang J., Wu C.F. (1994). Improved stability of Drosophila larval neuromuscular preparation in haemolymph-like physiological solutions. J. Comp. Physiol. A.

[72] De Castro C., Titlow J., Majeed Z.R., Cooper R.L. (2014). Analysis of various physiological salines for heart rate, CNS function, and synaptic transmission at neuromuscular junctions in Drosophila melanogaster larvae. J. Comp. Physiol. A.

[73] Iwaya A., Nakagawa S., Iwakura N., Taneike I., Kurihara M., Kuwano T., Gondaira F., Endo M., Hatakeyama K., Yamamoto T. (2005). Rapid and quantitative detection of blood Serratia marcescens by a real-time PCR assay: Its clinical application and evaluation in a mouse infection model. FEMS Microbiol. Lett..

